# Cascade Enhancement and Efficient Collection of Single Photon Emission under Topological Protection

**DOI:** 10.1021/acs.nanolett.4c03588

**Published:** 2024-09-19

**Authors:** Yali Jia, Zhaohua Tian, Qi Liu, Zhengyang Mou, Zihan Mo, Yu Tian, Qihuang Gong, Ying Gu

**Affiliations:** † State Key Laboratory for Mesoscopic Physics, Department of Physics, Peking University, Beijing 100871, China; ‡ Frontiers Science Center for Nano-optoelectronics & Collaborative Innovation Center of Quantum Matter & Beijing Academy of Quantum Information Sciences, Peking University, Beijing 100871, China; § Collaborative Innovation Center of Extreme Optics, Shanxi University, Taiyuan, Shanxi 030006, China; ∥ Peking University Yangtze Delta Institute of Optoelectronics, Nantong 226010, China; ⊥ Hefei National Laboratory, Hefei 230088, China

**Keywords:** Cascade Purcell enhancement, Efficient collection, High quantum yield, Topological protection

## Abstract

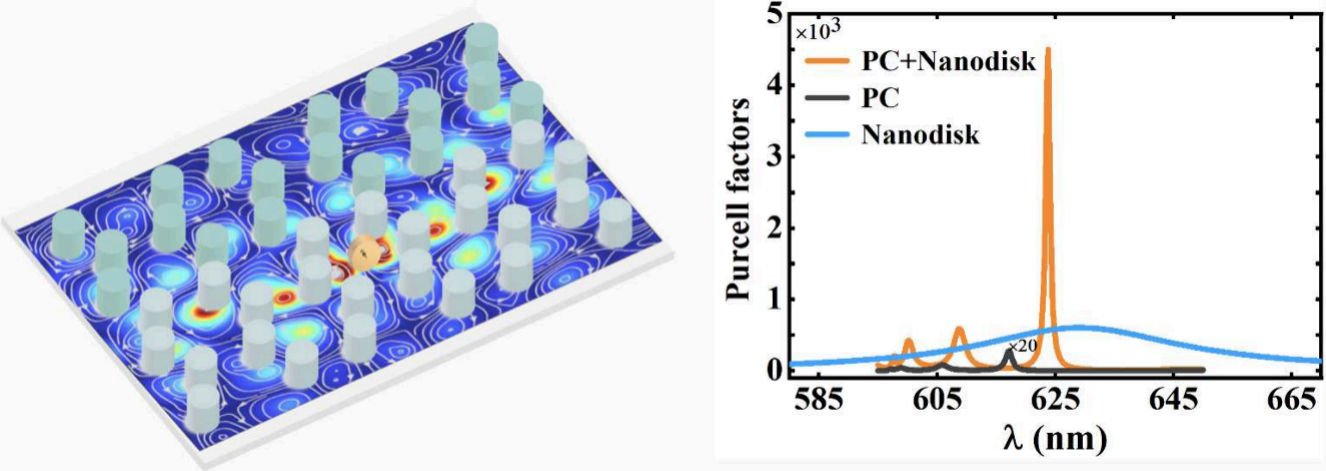

High emission rate, high collection efficiency, and immunity to defects are the requirements of implementing on-chip single photon sources. Here, we theoretically demonstrate that both cascade enhancement and high collection efficiency of emitted photons from a single emitter can be achieved simultaneously in a topological photonic crystal containing a resonant dielectric nanodisk. The nanodisk excited by a magnetic emitter can be regarded as a large equivalent magnetic dipole. The near-field overlapping between this equivalent magnetic dipole and edge state enables achieving a cascade enhancement of single-photon emission with a Purcell factor exceeding 4 × 10^3^. These emitted photons are guided into edge states with a collection efficiency of more than 90%, which is also corresponding to quantum yield due to topological antiscattering and the absence of absorption. The proposed mechanism under topological protection has potential applications in on-chip light–matter interactions, quantum light sources, and nanolasers.

Single photon sources in micro- and nanoscale are essential for on-chip quantum information processing. Enhancement of spontaneous emission through the optical modes in cavities, i.e., the Purcell effect, is one of the basic principles for realizing single photon sources.^1^ High photon emission rate, high collective efficiency, high quantum yield, and robustness to defects and perturbations are essential elements for the practical single photon sources.^2,3^ In order to meet the requirements above, various micronano photonic structures supporting diverse optical modes have been proposed. Both photonic crystal (PC) cavities by confining light in defects^4,5^ and PC waveguides with slow light near the photonic band edge^6,7^ enhance the spontaneous emission. But owing to large mode volume, the maximum enhancement of the emission rate is only several tens of γ_0_,^8^ where γ_0_ is the spontaneous emission rate in a vacuum. Although there is a high collection efficiency of emitted photons in the PC waveguide,^9−11^ a low emission rate makes it difficult to be used in on-chip photonic devices. The hotspots induced by surface plasmon polaritons (SPPs) in metallic nanoparticles can achieve a high emission rate of more than 10^4^γ_0_,^12−16^ while its radiation part accounts for a small proportion due to the large absorption. There is higher extraction efficiency in the SPP waveguide,^17−19^ but the large absorption loss leads to low quantum yield, hindering its application. In addition, dielectric structures overcoming the loss problem and supporting multipole resonances can increase the enhancement of electric and magnetic emission up to several hundreds of γ_0_, but the emitted photons are difficult to collect and utilize.^20−24^

In order to achieve both high emission rate and collection efficiency, hybrid micro- and nanostructures with abundant near-fields are considered.^25−28^ Because the SPP structure has a smaller mode volume, to obtain superior properties, it becomes a good candidate to be combined with other micronano structures. First, owing to the ultrasmall mode volume at the nanoscale gap, the gap SPP structure provides large spontaneous emission enhancement.^29,30^ By combining it with low-loss optical fiber, ultrahigh photon emission rate and one-dimensional nanoscale photon propagation are simultaneously obtained.^31^ Second, joining an SPP structure with a high-quality factor whispering gallery mode not only has strong emission enhancement^32,33^ but also has a mixed-mode line width tunability.^34^ Third, in a hybrid structure of PCs and SPPs, the local field can be greatly enhanced,^35^ so that a large Purcell factor can be achieved.^36,37^ From the above hybrid structures, one can see that, due to the existence of SPPs, a large emission rate can be obtained, but the loss of metal materials makes the quantum yield low. Moreover, the properties of these micronano cavities are affected by defects and disturbances during the manufacturing process, which further limits their application in high-quality single photon sources.

The unique properties of topological photonics, including topological robustness and antiscattering, bring new opportunities for the development of single photon sources.^38−42^ Barik et al. first proposed the chiral coupling between quantum emitters and edge states in topological PCs.^43^ After that, Purcell enhancement is demonstrated with topological corner states,^44,45^ topological slow light in valley PCs,^46,47^ and topological Su–Schrieffer–Heeger cavity modes.^48^ Unfortunately, their Purcell factor is not very high, almost on the same order as that in photonic crystals. Recently, nonscattering large Purcell enhancement is obtained in the topological photonic structure containing a plasmonic nanoantenna,^49^ but still suffers from low quantum yield due to an intrinsic metallic loss. So, that both high photon emission rates and high quantum yield simultaneously exist under topological protection remains an issue.

In the following, by combining robust edge states in a topological PC with magnetic dipole resonance in a low-loss dielectric nanodisk, taking advantage of both, we theoretically achieve cascade enhancement and high quantum yield of single photon emission under topological protection (Figure 1a). The resonance of the nanodisk excited by a magnetic emitter can be regarded as a large equivalent magnetic dipole (Figure 1c). Superior to metallic nanoparticles, this nanodisk has a large Purcell factor but without any nonradiative part. Fortunately, there is large near-field overlapping between this equivalent magnetic dipole and topological edge state appearing at the interface of two PCs. As a result, we achieve a cascade enhancement of single photon emission with a Purcell factor exceeding 4 × 10^3^. Also owing to the near-field overlapping and topological robustness, the photons scattered around the nanodisk are guided into edge states with a collection efficiency of more than 90%. Since there is no absorption loss and the edge state has the property of antiscattering, in principle, the values of collection efficiency and quantum yield should be equal. The proposed mechanism for bright single-photon emission and high quantum yield under topological protection opens up the opportunities for practical applications of on-chip light–matter interaction, quantum light sources, and nanolasers.

**Figure 1:**
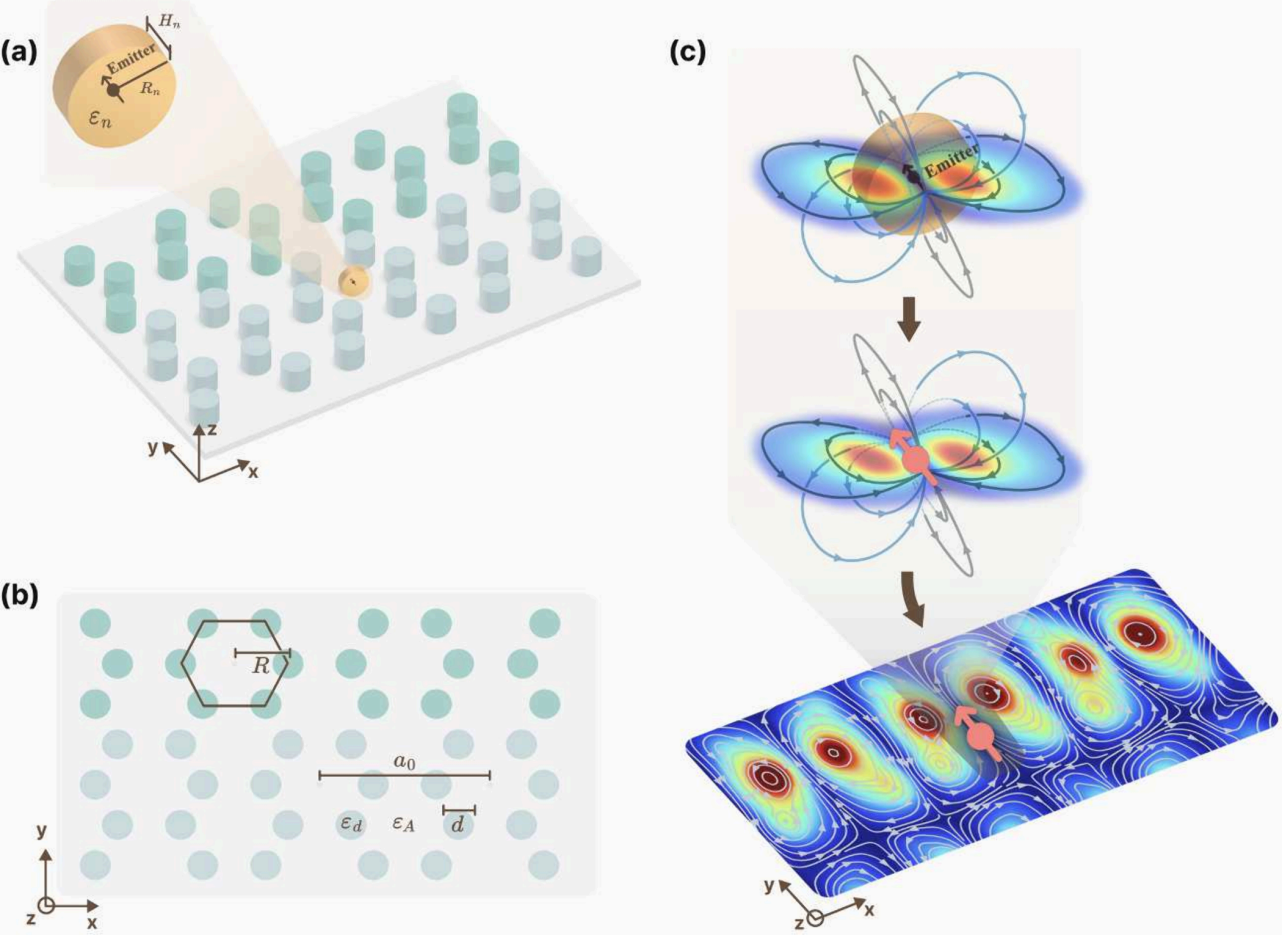
Scheme of cascade Purcell enhancement under topological protection. (a) Schematic diagram of the topological PC-resonant nanodisk hybrid structure. A resonant nanodisk with radius *R*_*n*_, height *H*_*n*_, and dielectric constant ε_*n*_ are inserted at the splices of PCs with different topological properties. The quantum emitter is located in the center of the nanodisk. (b) Schematic diagram of the honeycomb PC, where *R* is the length of the hexagonal side and *d* is the diameter of the dielectric cylinder. ε_*d*_ and ε_*A*_ are the dielectric constant of the dielectric cylinder and the surrounding environment, respectively. (c) The magnetic dipole resonance of the nanodisk excited by the magnetic quantum emitter (brown arrow) can be equivalent to a large magnetic dipole (pink arrow) interacting with the edge state. The streamlines around the nanodisk and the equivalent magnetic dipole are magnetic field lines. The bottom is the electric field distribution of the edge state, and the white streamlines are the magnetic field lines.

Edge states with robust and antiscattering properties are supported in topological PCs. However, the Purcell enhancement is only several tens of γ_0_ in the bare topological PC due to the relatively extended mode volume of the edge states. This limits its application in efficient quantum information processing.^46,47^ Low-loss dielectric nanostructures support magnetic dipole resonance with a small mode volume.^50^ However, photons scattered around are difficult to collect and utilize. Therefore, here we combine the advantages of both and design a hybrid structure of topological PC containing a resonant nanodisk (Figure 1a). Cascade enhancement of emission and efficient collection of emitted photons are achieved simultaneously.

In the topological hybrid structure, the quantum emitter is placed in the near-field region of the nanodisk, and the total decay rate can be divided into two parts, γ_tot_ = γ_ed_ + γ_sc_, where γ_ed_ is the part that decays to the edge state and γ_sc_ is the part that radiates into the free space. For a nanodisk in free space, all photons are scattered into free space, i.e., γ_tot_ = γ_sc_. In the following, we use COMSOL Multiphysics software to calculate the Purcell factor (defined as PF = γ/γ_0_) for each part. The calculation details are given in the Supporting Information.

Consider the hybrid structure of a topological PC containing a resonant nanodisk (Figure 1a), where the honeycomb topological PC with *C*_6_ symmetry was proposed by Hu’s group.^51^ The primitive cell of PC is shown in Figure 1b, which is a hexagonal honeycomb composed of six cylinders. Here *a*_0_ is the lattice constant and *R* is the distance from the center of the primitive cell to the center of the dielectric cylinder. In the cell, the diameter of the dielectric cylinder is *d* = 65 nm and the dielectric constant is ε_*d*_ = 11.7, and the rest is air with the dielectric constant ε_*A*_ = 1. When the lattice constant *a*_0_ is fixed at 319 nm, *R* is adjusted. When *a*_0_/*R* = 3.3, it is a topologically trivial PC (the upper PC in Figure 1b). When *a*_0_/*R* = 2.7, it is a topologically nontrivial PC (the lower PC in in Figure 1b). By splicing two photonic crystals with different topological properties together, edge states appear at about λ = 595–650 nm. In the edge state channel, a dielectric nanodisk with a dielectric constant of ε_*n*_ = 16, a radius of *R*_*n*_ = 75 nm, and a height of *H*_*n*_ = 89 nm is inserted, with its axial direction being the *y*-axis. The nanodisk supports a magnetic dipole resonance with λ = 629.2 nm, within the spectral range of the edge state. The *y*-polarized magnetic quantum emitter is located at the center of the nanodisk in the topological hybrid structure. We used COMSOL Multiphysics software to simulate the spectral properties of edge states in topological PCs and the magnetic dipole resonance of the nanodisk (Supporting Information).

We now investigate the cascade enhancement of magnetic emission in the hybrid structure. As shown in Figure 2, when a quantum emitter is placed at the center of a nanodisk, the maximum magnetic Purcell factor γ_tot_/γ_0_ can reach 4504. While in bare topological PC, the maximum Purcell factor is only 14. When there is only a dielectric nanodisk, the Purcell factor at the resonance is 604. Therefore, much stronger emission enhancement can be achieved in the hybrid structure than in the bare topological PC and the bare dielectric nanodisk. To quantitatively describe the enhancement effect in the hybrid structure, we define the cascade enhancement factor  where PF_PC_, PF_Nanodisk_, and PF_Hybrid_ are the maximum Purcell factors in the bare topological photonic crystal, bare nanodisk, and hybrid structure, respectively. It can be seen that stronger emission enhancement can be achieved in the hybrid system than in nanodisk or topological PC alone, with the cascading effect. Here the cascade enhancement factor can reach δ = 0.53. If the cascade enhancement factor is 1, then the cascade enhancement effect is perfectly achieved. However, it is difficult for the cascade factor to arrive at 1 due to insufficient near-field overlapping between the equivalent magnetic dipole and the edge state. The larger the cascade enhancement factor, the better the cascade enhancement effect, that is, the higher the Purcell factor in the hybrid structure.

**Figure 2:**
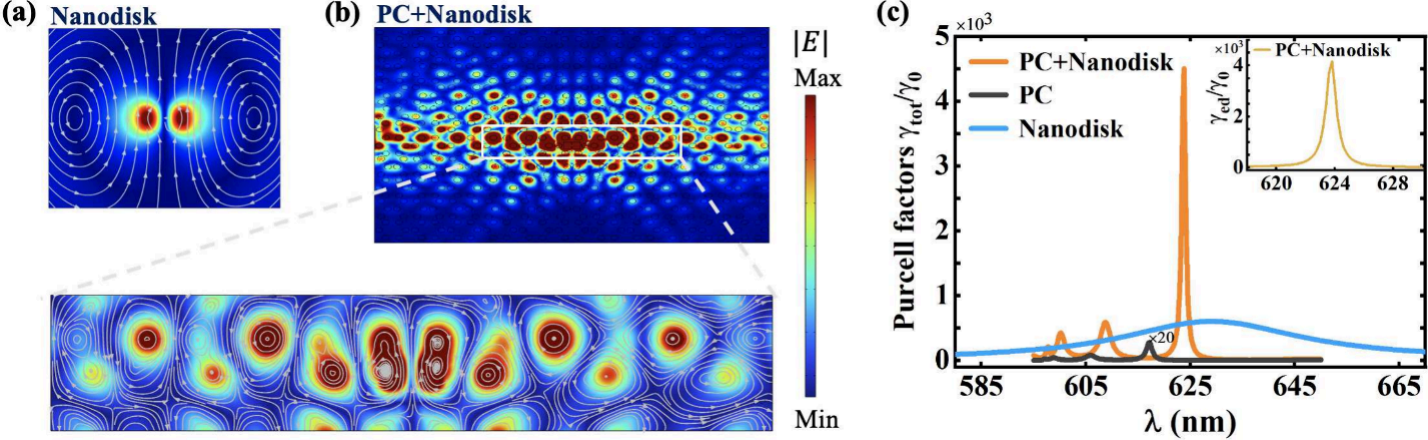
Cascade enhancement of emission and efficient collection of photons in topological hybrid structures. (a) The electric field in the *xy* plane of the magnetic dipole resonance in a bare nanodisk, where the white streamlines indicate the direction of the magnetic field. (b) The electric field of the edge state in the *xy* plane of the topological hybrid structure (top part). The region where the edge state is plotted is 6*a*_0_ × 1.2*a*_0_, where the white streamlines indicate the direction of the magnetic field (bottom part). (c) The total Purcell factors γ_tot_/γ_0_ in the bare nanodisk, bare topological PC, and the hybrid structure. The inset is the Purcell factor γ_ed_/γ_0_ along the edge state propagation in the hybrid structure. Purcell cascade enhancement can be achieved in the hybrid structure, with a cascade enhancement factor of up to 0.53. The efficiency of collecting photons along the edge states can reach 93%.

The mechanism of achieving magnetic emission cascade enhancement is described as follows. The magnetic dipole resonance supported by a dielectric nanodisk (Figure 2a) can be effectively excited by a magnetic emitter, which can be equivalent to a large magnetic dipole (Figure 1c). This large magnetic dipole and the edge state have magnetic fields with similar rotational characteristics and near-field overlapping (Figure 2b), so the magnetic emission cascade enhancement is achieved. The magnetic Purcell factor in topological hybrid structure is much larger than that in the dielectric structure proposed in ref (52). It should be noted that we focus on magnetic emission rather than electric emission. This is because the electric resonance has an electric field with rotational characteristics, while the electric field of the edge state of the TM mode does not have such characteristics; therefore, the near-field overlapping is not large.

For practical application of single photon sources, besides a large emission rate, efficient collection of photons is also extremely important. For a nanodisk in free space, emitted photons are difficult to collect and utilize. In the topological hybrid structure, the topological PC can guide the photons that were previously scattered to the surroundings into the edge states. If the collection efficiency is defined as β = γ_ed_/γ_tot_, β can reach 93% (inset in Figure 2c). The high collection efficiency in the hybrid structure comes from the large near-field overlapping between an equivalent large magnetic dipole and the edge state as well as antiscattering of topological state (Figure 2b). In previously reported enhanced emission via hybrid structures containing a metallic nanoantenna,^32−34^ the quantum yield is not very high. But here, owing to no absorption loss in dielectric structures and antiscattering property of edge states, the quantum yield is almost equal to the collection efficiency; i.e., almost all the emitted photons can be used for on-chip photonic devices. It is worth noting that the narrow line width in the hybrid structure also comes from the fact that edge states can collect scattered photons.^53^

To quantitively describe the near-field overlapping between the magnetic dipole resonance of the nanodisk and topological edge state, we define the overlapping degree as , where , , and  are the electric fields of the edge state, the resonant nanodisk without the PC, and the system background, respectively, and *V*_*m*_ is the calculation area (Supporting Information). By rotating the nanodisk around the *x*-axis by an angle θ (inset in Table 1), it can be seen that as θ increases, their near-field overlapping degree η decreases (Table 1). When θ becomes larger, the polarization of the equivalent large magnetic dipole is shifted, and then the number of photons to excite the edge state is smaller, i.e., their near-fields overlap less. As a result, there is a decrease of the cascade enhancement factor δ as well as the magnetic Purcell factor γ_tot_/γ_0_. Meanwhile, the efficiency of collecting photons along the edge states also becomes lower.

**Table 1:**
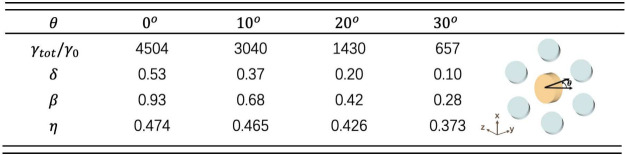
Purcell Factor γ_tot_/γ_0_, Cascade Enhancement Factor δ, Collection Efficiency β, and Near-Field Overlapping Degree η When the Nanodisk Is Rotated around the *x*-Axis by an Angle θ (Inset)^a^

a The parameters of the nanodisk are ε_*n*_ = 16, *R*_*n*_ = 75 nm, *H*_*n*_ = 89 nm. As θ increases, γ_tot_/γ_0_, δ, and β all decrease due to the decrease of the near-field overlapping η between the dipole resonance of the nanodisk and the edge state.

We now discuss the effects of the structural parameters of nanodisks on the cascade enhancement and collection efficiency of single photon emission. As the height *H*_*n*_ increases, both the Purcell factor and the cascade enhancement factor become larger (Figure 3a). Then, we find that the expansion of the radius *R*_*n*_ also leads to a higher Purcell factor and cascade enhancement factor (Figure 3b). The reason is that larger nanodisks have more near-field overlap with edge states (inset in Figure 3). In addition, when the size of the nanodisk is enlarged, the wavelength of magnetic dipole resonance red-shifts, so the spontaneous emission spectrum in the hybrid structure also red-shifts. It should be noted that the collection efficiency is insensitive to the size of the nanodisk, remaining almost constant. Here, the *y*-polarized magnetic quantum emitter located in the center of the nanodisk is used. Generally, the line width of the quantum emitter frequency is much less than the spectral width of the resonance in the hybrid structure. In this case, an equivalent magnetic dipole can fully excite the edge states. Moreover, the insensitivity of the collection efficiency to the size of the nanodisk comes from the robustness of the edge states.

**Figure 3:**
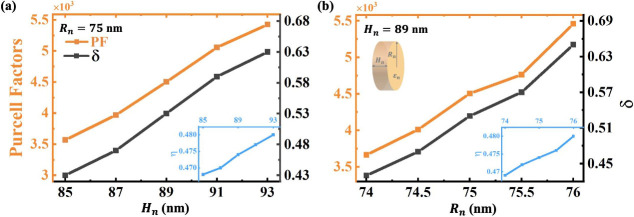
Influence of nanodisk size on the response of a topological hybrid structure. Purcell factor, cascade enhancement factor, and near-field overlapping (a) with different heights *H*_*n*_ of nanodisks and (b) with different radius *R*_*n*_ of nanodisks. As the size of the nanodisk increases, the near-field overlapping increases, so the Purcell factor and the cascade enhancement factor increase. Here, the dielectric constant is ε_*n*_ = 16.

In the topological hybrid structure, as long as the *y*-polarized magnetic quantum emitter is placed in the near-field region of the nanodisk, it can excite the magnetic dipole resonance, thereby obtaining a cascade enhancement of the Purcell factor. Since the magnetic hotspot is at the center of the disk, the maximum Purcell factor can be obtained when the quantum emitter is placed at the center, compared with the cases far away from the hot spot (Supporting Information). In addition, even if the position of the nanodisks in the topological PC is slightly shifted, the emission cascade enhancement phenomenon can still be observed (Supporting Information), which facilitates the experimental realization. During the experimental fabrication process, structural factors of a topological PC should also be considered. It is found that when the position and size of the dielectric cylinder in the topological photonic crystal change slightly, there is almost no effect on the Purcell factor and collection efficiency. Also, when the lattice constant of the topological photonic crystal increases, the spontaneous emission spectrum red-shifts, but the cascade enhancement effect and high collection efficiency can still be achieved (Supporting Information). Thus, our proposed mechanism for emission cascade enhancement and efficient collection is robust to structural disorder due to topological protection.

The magnetic emission cascade enhancement is sensitive to the polarization of the quantum emitter. Only when the magnetic dipole resonance of the nanodisk is sufficiently excited does the large near-field overlapping with the edge states occur, resulting in a cascade enhancement of the emission. Since the *x*-polarized and *z*-polarized dipoles excite the magnetic dipole resonance of the nanodisk insufficiently, there is no large emission enhancement (Supporting Information). This mechanism of emission enhancement has potential applications in on-chip single-photon sources. When the structural defect is created by removing the cylinder from the PC, the cascade enhancement factor and collection efficiency are almost the same as those without the defect (Supporting Information). This topological protection is beneficial for experimental implementation.

In such a hybrid structure, a stronger local field enhancement can be produced than in the edge state or nanodisk alone; that is, the enhancement of the near field is also a cascade effect. This is because the large near-field overlapping between the nanodisk and the edge state enables the energy originally scattered into free space to be collected by the edge state channel, thus enhancing the near-field of the nanodisk in the hybrid structure. The mechanism of emission cascade enhancement under topological protection is possible to implement at infrared and microwave frequencies. This requires a magnetic dipole resonance and edge state engineered at infrared or microwave frequencies, while the sizes of dielectric nanoparticles and topological photonic crystals are compatible. Resonant nanoparticles and topological edge states have been demonstrated in infrared and microwave spectra.^43,55,63,64^

Our proposed mechanism for cascade enhancement and efficient collection of single-photon emission will have a profound impact on other hybrid structures. The emission cascade enhancement effect can be extended to other similar topological hybrid structures. For example, a nanodisk can be replaced with nanoantennas, nanospheres, and other micronanostructures that support dielectric resonance.^65^ The edge states supported by a topological photonic crystal can also be replaced by topological modes in other topological structures, such as edge states in topological valley photonic crystals, topological corner states, etc.^66^ For other types of hybrid structures, if there is a large near-field overlapping between the modes, emission cascade enhancement may also be achieved but without topological protection. Then, the efficient collection of photons arises from the robust edge states. If the edge states are replaced by other guided modes, such as waveguide modes in nanowires.^67^ the collection efficiency may be reduced. Moreover, the emission enhancement effect is not limited to magnetic emission but is also applicable to electric emission. Theoretically, the electric resonance of nanoparticles can be equivalent to a large electric dipole. If a large near-field overlap with the electrical resonance of the nanoparticles can be achieved, then cascade enhancement of the electrical emission is also possible.

Finally, we discuss the possibility of the experimental implementation of our scheme. At present, both topological PC^54^ and dielectric nanocavities^55^ can be manufactured using nanotechnology, e.g., electron beam lithography and electrochemical etching. Single emitters can be realized in many forms, such as molecules,^56^ Rydberg atoms,^57^ and quantum dots.^58^ In some quantum emitters such as rare earth ions^59^ and semiconductor quantum dots,^60^ there are significant magnetic dipole transitions whose intensities are comparable to or even stronger than the competing electric dipole transitions. It is possible to place emitters into the hybrid topological structure, such as by integrating semiconductor quantum dots into topological waveguides^61^ or doping rare-earth ions into nanodisks.^62^ Therefore, the scheme that we proposed here may be experimentally realized in the future.

In summary, we have proposed a mechanism for the cascade enhancement and efficient collection of emitted photons under topological protection. A hybrid structure integrating a topological PC and nanodisk is designed in which the magnetic dipole resonance of the nanodisk can be equivalent to a large magnetic dipole to interact with the edge state. The large near-field overlapping between the resonant nanodisk and the edge state enhances the magnetic emission through a cascade effect. At the same time, emitted photons can be efficiently guided to propagate in the edge state channel, achieving a collection efficiency exceeding 90% due to the robustness of the edge state. The low loss of the dielectric structure and the antiscattering properties of the topological mode make the quantum yield almost equivalent to the collection efficiency; that is, almost all of the emitted photons can be used in on-chip photonic devices. The mechanism proposed here enhances the spontaneous emission rate of the emitter while achieving large collection efficiency under topological protection, which will provide practical use for ultrabright and stable on-chip single photon sources. It also provides new insights for the study of cavity quantum electrodynamics in topological structures.

## Supporting Information Available

Topological photonic crystals, magnetic dipole resonance of nanodisk, the calculation of Purcell factor, collection efficiency, calculation of near-field overlapping degree, effect of emitter polarization and position on Purcell factors, effect of nanodisk position on the Purcell factors, the robustness of cascade enhancement and efficient collection, and effects of size and position of topological photonic crystal structures on the Purcell factors (PDF)
