# Supplementary material for: Cascade Enhancement and Efficient Collection of Single Photon Emission under Topological Protection
Source: Nano Lett. 2024 Sep 19;24(39):12277–84. doi: 10.1021/acs.nanolett.4c03588 (PMC11451449; doi:10.1021/acs.nanolett.4c03588)
Supplement: Supplementary file 1 — nl4c03588_si_001.pdf [file nl4c03588_si_001.pdf]

Supplemental Material for Cascade enhancement and efficient collection of single photon emission under topological protection

Yali Jia [1], Zhaohua Tian[1], Qi Liu[1,2], Zhengyang Mou[1], Zihan Mo[1], Yu Tian[1,2], Qihuang Gong[1,2,3,4,5], and Ying Gu[1,2,3,4,5]*

[1]*State Key Laboratory for Mesoscopic Physics, Department of Physics, Peking University, Beijing 100871, China*
[2]*Frontiers Science Center for Nano-optoelectronics & Collaborative Innovation Center of Quantum Matter & Beijing Academy of Quantum Information Sciences, Peking University, Beijing 100871, China*
[3]*Collaborative Innovation Center of Extreme Optics, Shanxi University, Taiyuan, Shanxi 030006, China*
[4]*Peking University Yangtze Delta Institute of Optoelectronics, Nantong 226010, China*
[5]*Hefei National Laboratory, Hefei 230088, China*

(Dated: September 5, 2024)

1. TOPOLOGICAL PHOTONIC CRYSTAL

1.1 Photonic bands

Xiao hu's group proposed that two-dimensional topological photonic crystal (PC) based on dielectric materials can be obtained by deforming the honeycomb lattice [1]. Fig. S1(a) shows the schematic diagram of the topological PC, which consists of a hexagonal artificial atom composed of six cylinders made of dielectric material. By shrinking and expanding the honeycomb lattice, the doubly degenerate Dirac cone is opened to obtain topologically trivial and nontrivial band gaps. The contraction and expansion of the lattice are quantified by a_0/R, where a_0 is the lattice constant and R is the distance between cylinder's center and the unit cell's center. Here we consider the harmonic TM mode of electromagnetic waves in the PC, that is, there are out-of-plane E_z and in-plane H_x and H_y components with others being zero. We calculated the photonic band diagram under different a_0/R using COMSOL software, as shown in Fig. S1(b). When the lattice shrinks to $a_0/R = 3.3$, a photonic band gap opens, forming a topologically trivial structure. When $a_0/R = 3$, the Dirac point appears at Γ point. When the lattice expands to $a_0/R = 2.7$, the band gap is reopened with band inversion, forming a topologically nontrivial structure. Fig. S1(c) shows the photonic band diagram of a supercell consisting of PCs with parameters $a_0/R = 2.7$ and $a_0/R = 3.3$. Two dispersion curves representing edge states appear in the band gap. The band with a positive slope represents the pseudospin-up state, while the negative slope represents the pseudospin-down state [Fig. S1(d)].

1.2 Simulation of edge states

We use COMSOL multiphysics software to simulate the spectral properties of edge states in topological PC. Here, two PCs with different topological properties are spliced to build 2D and 3D modules of topological PCs.

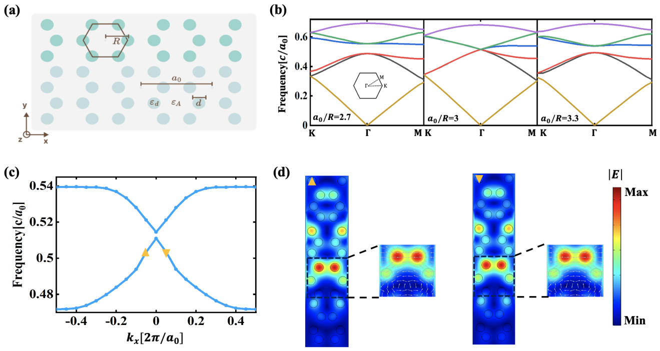

FIG. S1. (a) Schematic diagram of a 2D honeycomb photonic lattice. (b) Photonic band diagrams of PCs with different parameters a_0/R. (c) Photonic band diagram of a supercell composed of PCs with parameters a_0/R=3.3 and a_0/R=2.7. (d) The electric field distribution of the edge states at the triangles in (c), where the white arrow indicates the direction of energy flow

The module includes 12×16 unit cells. In the 2D module, scattering boundary conditions are used for the surrounding boundaries. In the 3D module, the height of the cylinder is $h = 300$ nm. Perfect electric conductor boundary conditions are applied to the top and bottom xy planes of the entire structure to mitigate the electrical response's influence. Scattering boundary conditions are used in the other boundaries. Transmission spectra were calculated under plane wave excitation for both 2D and 3D modules [Figs. S2(a) (b)]. Photons can propagate through the edge states resulting in a high transmittance in the band gap. Figs. S2(c) (d) show the electric field of the edge states. The transmittance decreases near $\lambda = 630$ nm because there is a small gap in the dispersion relationship of the edge state in Fig. S1(c). The transmission spectra of edge state in the 2D module are consistent with those in the 3D module.

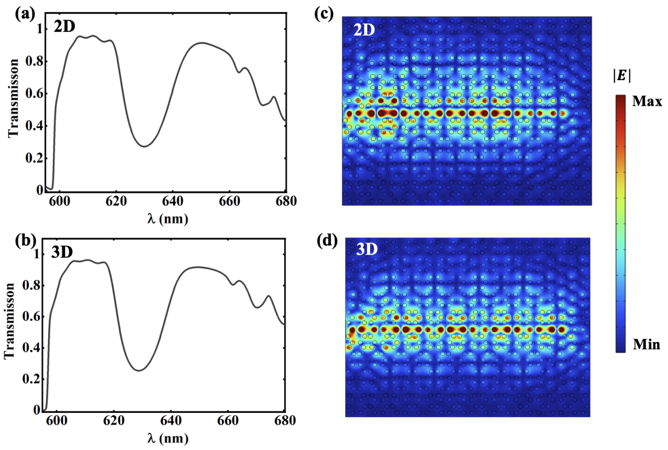

FIG. S2. Transmission spectra of edge states in (a) 2D and (b) 3D modules of topological PC. Electric fields of edge states in (c) 2D and (d) 3D modules of topological PC.

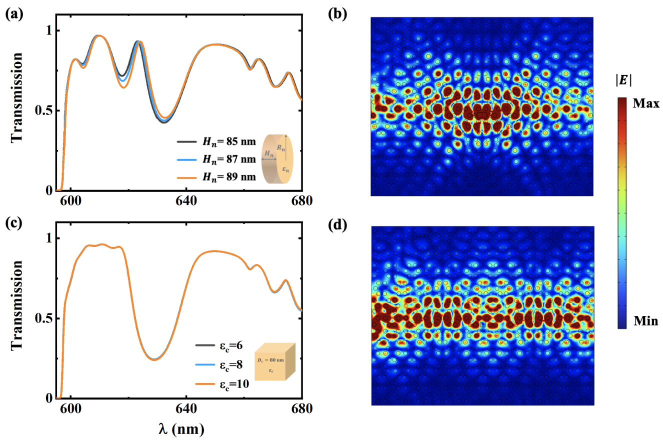

FIG. S3. (a) Transmission spectra and (b) electric field distributions of edge states when nanodisks of different heights are placed into a topological PC. (c) Transmission spectra and (d) electric field distributions of edge states when cubes with different dielectric constants are placed in a topological PC.

1.3 Robustness of edge states

The topological mode is robust to defects and impurities [2]. Here, we demonstrate the robustness of edge states by placing nanoparticles of different shapes in a topological PC. Nanodisks of different heights are embedded in a topological PC. In this case, the transmission spectrum still has high transmittance in the frequency range of the band gap [Fig. S3 (a)]. The electric field distribution of the edge state is almost unchanged compared to the topological structure without impurities [Fig. S3 (b)]. Other parameters of the nanodisk are $\varepsilon_n = 16$ and R_n=75 nm. In another case, we alter the shape of the impurity nanoparticles to cubic and modify their dielectric constants, with a side length of $D_c = 80$ nm. The Figs. S3 (c) (d) show the transmission spectrum and the electric field distribution of the edge state, which are almost consistent with the situation in the topological PC without impurities. The edge states are almost not affected by impurities, resulting in high transmittance even if the structure is defective.

2. MAGNETIC DIPOLE RESONANCE OF NANODISK

We study the magnetic dipole resonance of nanodisk in free space via COMSOL software. A nanodisk is embedded within a vacuum square with a side length of 3λ, where the scattering boundary condition is used to reduce reflection at the boundaries. The y-polarized magnetic dipole is placed at the center of the nanodisk. The parameters of the nanodisk are $R_n = 75$ nm, $H_n = 89$ nm and $\varepsilon_n = 16$. The Purcell factor is about 604 at the magnetic dipole resonance wavelength of 629.2 nm. As its indicated, the multipole expansion of the resonant electromagnetic field is equivalent to a y-polarized magnetic dipole. This can be identified from the field distribution and streamlines of the model [Fig. S4(c)]. As the height H_n increases, the magnetic dipole resonance redshifts and the Purcell factor increases slightly [Fig. S4 (a)]. We also calculate the Purcell factor as the nanodisk rotates around the x-axis by an angle θ, while its structural and material parameters as well as the polarized direction of the excitation magnetic dipole are fixed. A larger angle results in a smaller Purcell factor [Fig. S4(b)]. The variation with angle does not affect the resonance wavelength.

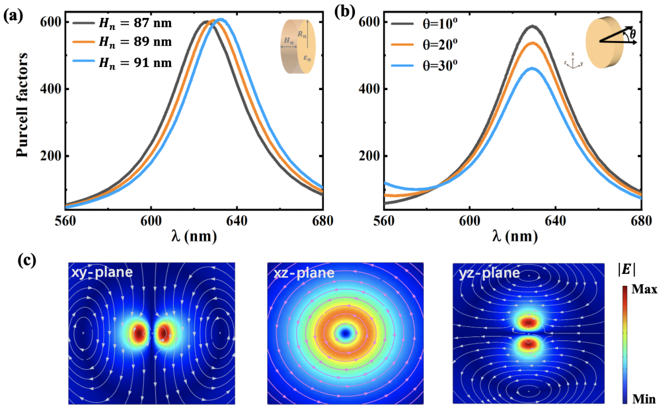

FIG. S4. (a) Purcell factors with different heights of nanodisk, the other parameters of the nanodisk are $R_n = 75$ nm and $\varepsilon_n = 16$. (b) Purcell factors for a nanodisk with $R_n = 75$ nm, $H_n = 89$ nm and $\varepsilon_n = 16$ rotating around the x-axis at an angle θ. (c) Electric field distributions of the magnetic dipole resonance of a nanodisk in the xy, xz and yz planes. Gray streamers and arrows indicate the direction of the local magnetic field. Pink streamers and arrows indicate the direction of the local electric field.

3. THE CALCULATION OF PURCELL FACTOR

We describe the details of the calculation of Purcell factor in topological hybrid structure. Here, the total decay rate is divided into two parts $\gamma_{tot} = \gamma_{ed} + \gamma_{sc}$, where γ_{ed} is the part that decays to the edge state and γ_{sc} is the part that decays into free space. The Purcell factors for each part are calculated from the ratio of these decay rates to the vacuum decay rate (γ_{tot}/γ_0, γ_{ed}/γ_0,γ_{sc}/γ_0). The Purcell factors for each part of the quantum emitter can be obtained from $\gamma_i/\gamma_0 = W_i/W_0(i = ed, sc)$, so the simulation of the decay rate can be transformed into the simulation of energy [3, 4]. W_0 is the energy emitted by emitter placed in free space, while W_{tot} denotes the total energy emitted by emitter embedded in the topological hybrid structure. Both values are obtained through surface integrals of the energy flows around the nanosphere that encloses the emitter. The fraction of W_{ed} propagating along the topological edge state is obtained by surface integrals over energy flows in the propagation direction. The other scattering part, W_{sc} is obtained by surface integrals over energy flows on the other boundaries.

4. COLLECTION EFFICIENCY

In topological hybrid structures, topological PC can collect photons scattered around through edge state channel. The collection efficiency is defined as $\beta = \gamma_{ed}/\gamma_{tot}$, where γ_{ed} is the fraction that propagates along the edge states and γ_{tot} is the total emission rate. As in Section 3, the conversion of decay rate into energy is simulated in COMSOL software. Here, receiving surfaces of different size in a topological PC are used to calculate the energy W_{ed} propagating along edge states [inset of Fig. S5]. The values of γ_{ed}/γ_0 obtained under different receiving surface sizes are almost the same, about 4000 [Fig. S5]. This is because edge states are localized at the interface where PCs with different topological properties are spliced.

The Table I shows the variation in collection efficiency when nanodisks of different sizes are embedded in a topological PC. It can be seen that the efficiency of collecting photons along the edge states remains almost constant when varying the size of the nanodisk. The collection efficiency is insensitive to the size of the nanodisk.

5. CALCULATION OF NEAR-FIELD OVERLAPPING DEGREE

Both the cascade enhancement of emission and the efficient collection of emitted photons in the topological hybrid structure originate from the large near-field overlapping between the magnetic dipole resonance of the nanodisk and the edge states of the topological PC. For

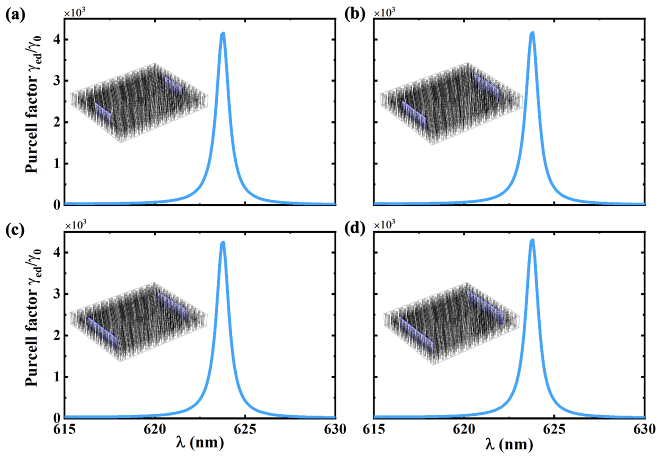

FIG. S5. The influence of receiving surface size (a) $3a_0 \times h$, (b) $4a_0 \times h$, (c) $5a_0 \times h$, and (d) $6a_0 \times h$ on the Purcell factor γ_{ed}/γ_0. The receiving surface is shown in the inset.

quantitative analysis, we define the near-field overlapping degree as $\eta = \int \frac{|\vec{E}_1 \cdot \vec{E}_2|}{E_0^2 V_m} dV$ [6], where $\vec{E}_1$ is the electric field of the edge state, $\vec{E}_2$ is the electric field of the resonant nanodisk without topological PC structure, $\vec{E}_0$ is the electric field of the system background, and V_m is the calculated area. The computational region is divided into many meshes with the number N. The near-field overlapping degree can be transformed into $\eta = \sum_{i=1}^{N} \frac{|\vec{E}_{1,i} \cdot \vec{E}_{2,i}|}{E_0^2 N}$, where $\vec{E}_{1,i}$ and $\vec{E}_{2,i}$ are the electric field of edge state and resonant nanodisk without PC in the ith single mesh, respectively.

6. EFFECTS OF EMITTER'S POLARIZATION AND POSITION ON PURCELL FACTORS

First, we discuss the effect of the polarization of the emitter on the magnetic Purcell factor. In the main text, the polarization of the magnetic emitter is along the y-axis, which is parallel to the axis of the nanodisk in the topological hybrid structure. We also calculated the Purcell factors under the excitation of x-polarized and z-polarized magnetic emitters, respectively. The param-

TABLE I. Variation of collection efficiency (β) with respect to the height (H_n) and radius (R_n) of the nanodisk. In (a), the radius is fixed as R_n=75 nm. In (b), the height is fixed as H_n=89 nm.

(a)	H_n	87 nm	89 nm	91 nm
	β	0.93	0.93	0.92
(b)	R_n	74.5 nm	75 nm	75.5 nm
	β	0.92	0.93	0.92

eters of topological PC and nanodisk are the same with those in the main text. It is found that large Purcell enhancement is obtained only when the system is excited by a y-polarized emitter. For the z-polarization [Fig. S6(a)] and x-polarization [Fig. S6(b)] cases, the Purcell factor is only about one hundred. This is mainly because the x-polarized and z-polarized emitters cannot excite the magnetic dipole resonance of the nanodisk, making it impossible to achieve the cascade enhancement of magnetic emission in the hybrid structure.

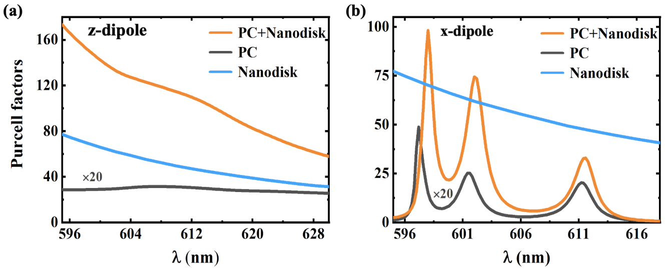

FIG. S6. Purcell factors in bare PC, bare nanodisk and hybrid structures under (a) z-polarized dipole and (b) x-polarized dipole excitation.

Next, we focus on the relationship between the position of the quantum emitter and the Purcell factor. In the main text, the quantum emitter is located at the center of the nanodisk. Here, the quantum emitter is moved by a distance d_e along the x-axis [inset of Fig. S7(a)]and the y-axis [inset of Fig. S7(b)] respectively. It can be found that the wavelength at the peak of the Purcell factor spectrum does not shift substantially. At the same time, the farther the quantum emitter is located from the center, the smaller the Purcell factor will be [Fig. S7]. This is because the magnetic dipole resonance of the nanodisk features a magnetic hot spot at its center. Therefore, when the quantum emitter is far away from the magnetic hot spot, a smaller Purcell factor is obtained.

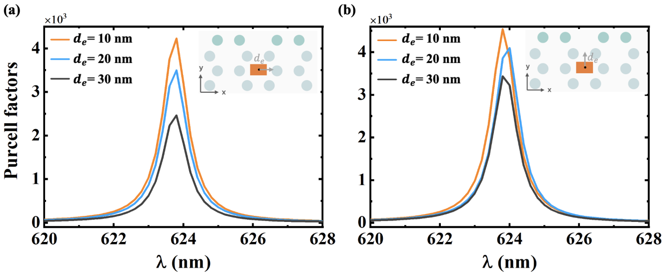

FIG. S7. The variation of the Purcell factors when the quantum emitter is moved by a distance d_e along the (a) x- and (b) y-axis directions. The inset is a schematic diagram of changing the position of the quantum emitter.

7. EFFECT OF NANODISK POSITION ON THE PURCELL FACTORS

In the main text, the nanodisk is located in the center of the PC, however it is difficult to embed the nanodisk accurately in the center of the PC in the experiment. Here, we discuss the change in the Purcell factor when the position of the nanodisk is slightly shifted. The nanodisk is moved along the x-axis [inset of Fig. S8(b)] and y-axis [inset of Figs. S8(c)(d)] by a distance d_s, where $d_s > 0$ means moving in the positive direction along the axis and $d_s < 0$ means moving in the negative direction along the axis. The magnetic emitter is located in the center of the nanodisk. For the case of a nanodisk shifted along the x-axis, the Purcell factor is almost constant [Fig. S8(b)]. As the nanodisk goes along the y-axis, the peak of the Purcell factor spectrum redshifts with increasing d_s, and can be maintained around 4500 as shown in Fig. S8(c)(d). Although the position of the nanodisk is slightly shifted, there is still a large near-field overlapping between the magnetic dipole resonance and the edge states, thus large Purcell enhancement can still be achieved.

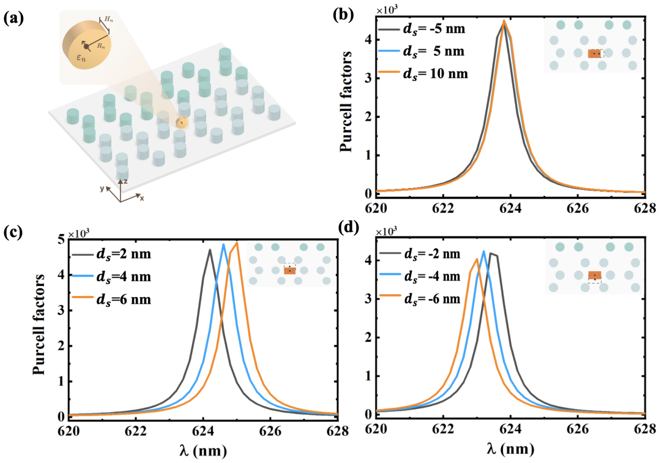

FIG. S8. (a) Schematic diagram of a resonant nanodisk embedded in a topological PC. Purcell factors with different positions of nanodisk. The nanodisk moves d_s along the (b) x-axis and along the y-axis in the (c) positive direction and the (d) negative direction, where $d_s > 0$ means moving to the positive direction along the axis and $d_s < 0$ means moving to the negative direction along the axis.

8. THE ROBUSTNESS OF CASCADE ENHANCEMENT AND EFFICIENT COLLECTION

Topological photonics offers a way to develop photonic devices that are robust to defects and disorder [5]. We removed PC cylinders at different locations to set up structural defects [inset of Fig. S9]. It can be seen that even

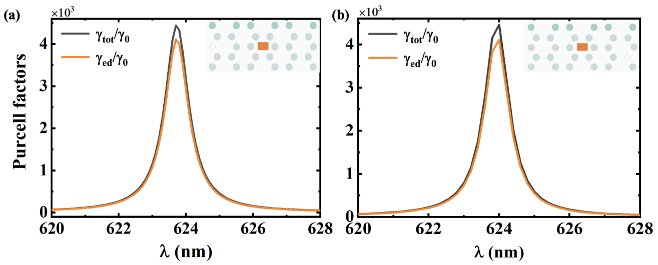

FIG. S9. The total Purcell factor γ_{tot}/γ_0 and the part γ_{ed}/γ_0 directed to edge states for different structural defects. The structural defect caused by removing the PC cylinder is shown in the inset.

if there are defects in the topological PC, the total Purcell factor γ_{tot}/γ_0 can reach more than 4400, of which the part guided into the edge state γ_{ed}/γ_0 is about 4100 [Fig. S9]. In the hybrid structure without defects, the purcell factors γ_{tot}/γ_0 and γ_{ed}/γ_0 are around 4500 and 4100, respectively. The cascade enhancement factor and collection efficiency are similar in the hybrid structures with and without defects. These results illustrate the robustness of cascade enhancement and efficient collection in hybrid structures, which facilitates experimental implementation.

9. EFFECTS OF SIZE AND POSITION OF TOPOLOGICAL PHOTONIC CRYSTAL STRUCTURES ON THE PURCELL FACTORS

We consider the effects of various factors in topological PC on emission cascade enhancement and efficient collection, including the size and position of the structure.

Firstly, we focus on the effect of the radius of the dielectric cylinder in the topological PC on the Purcell factor and collection efficiency. Here we enlarge the diameter of a dielectric cylinder to $d = 73$ nm, which is 8 nm larger than the other cylinders [inset of Fig. S10(a)]. It can be seen that the Purcell factor can still reach 4500, and more than 90% of the photons can be collected by edge state [Fig. S10(a)]. Fig. S10(b) shows that when the radius of the cylinder is reduced, there is almost no effect on the Purcell factor and collection efficiency. The position of the dielectric cylinder in the topological PC should also be considered. We calculated the Purcell factors γ_{tot}/γ_0 and γ_{ed}/γ_0 when a dielectric cylinder is moved 10 nm along the x-axis [inset of Fig. S10(c)] and y-axis [inset of Fig. S10(d)]. In this hybrid structure, the purcell factors γ_{tot}/γ_0 and γ_{ed}/γ_0 are around 4500 and 4100, respectively [Figs. S10(c)(d)].

Secondly, we study the case that the diameters of all cylinders in a unit cell of the PC structure were enlarged to $d = 69$ nm [inset of Fig. S11(a)], and the cylinders in the unit cell were shifted by 5 nm along the center of the hexagon [inset of Fig. S11(b)]. The results show

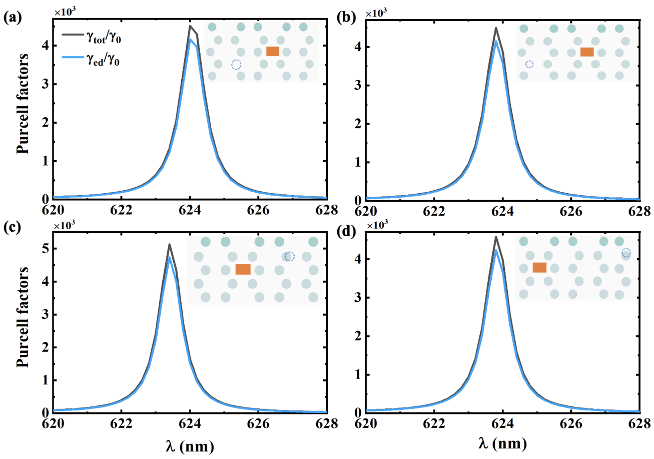

FIG. S10. Purcell factors when the diameter of a dielectric cylinder in topological PC is (a) enlarged and (b) reduced. Purcell factors when a dielectric cylinder in topological PC moves along the (c) x-axis and (d) y-axis. γ_{tot}/γ_0 is the total Purcell factor and γ_{ed}/γ_0 is the part that propagates along the edge state. The inset show the variation in size and position of a dielectric cylinder.

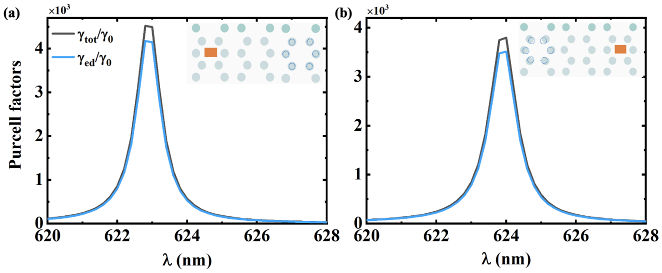

FIG. S11. Purcell factors when the (a) size and (b) position of all dielectric cylinders in a unit cell of a hybrid structure vary. The inset show the changes in the size and position of the dielectric cylinders in a unit cell.

that even in this case of structural disorder caused by the size and position of the dielectric cylinders, there is still emission cascade enhancement and efficient collection of photons [Fig. S11].

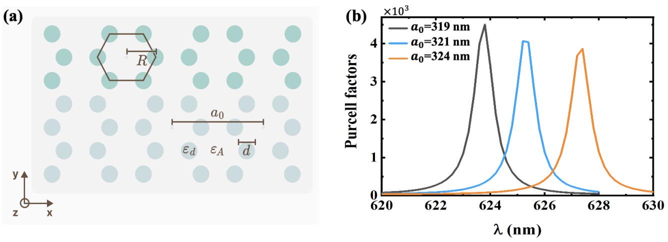

FIG. S12. (a) Schematic diagram of the topological PC. (b) The total Purcell factor varies with the lattice constant a_0.

Thirdly, we calculate the change in the Purcell factor as the size of the topological PC is increased, i.e., as

the lattice constant a_0 is increased. It can be seen that when the structure size increases, the spontaneous emission spectrum redshifts. Large Purcell factor of about 4000 and collection efficiency of around 90% can still be achieved in hybrid structure [Fig. S12].

* ygu@pku.edu.cn

[1] Wu, L.-H.; Hu, X. Scheme for achieving a topological photonic crystal by using dielectric material. Physical Review Letters 2015, 114, 223901.

[2] Tang, G.-J.; He, X.-T.; Shi, F.-L.; Liu, J.-W.; Chen, X.-D.; Dong, J.-W. Topological photonic crystals: physics, designs, and applications. Laser & Photonics Reviews 2022, 16, 2100300.

[3] Ren, J.; Gu, Y.; Zhao, D.; Zhang, F.; Zhang, T.; Gong, Q. Evanescent-vacuum- enhanced photon-exciton coupling and fluorescence collection. Physical Review Letters 2017, 118, 073604.

[4] Zhang, F.; Ren, J.; Shan, L.; Duan, X.; Li, Y.; Zhang, T.; Gong, Q.; Gu, Y. Chiral cavity quantum electrodynamics with coupled nanophotonic structures. Physical Review A 2019, 100, 053841

[5] Blanco-Redondo, A. Topological nanophotonics: toward robust quantum circuits. Proceedings of the IEEE 2019, 108, 837–849.

[6] Qian, Z.; Li, Z.; Hao,H.; Shan, L.; Zhang,Q.; Dong, J.; Gong, Q.; and Gu, Y. Absorption reduction of large purcell enhancement enabled by topological state-led mode coupling, Physical Review Letters 2021, 126, 023901.
